# Macroscopic Cluster Organizations Change the Complexity of Neural Activity

**DOI:** 10.3390/e21020214

**Published:** 2019-02-23

**Authors:** Jihoon Park, Koki Ichinose, Yuji Kawai, Junichi Suzuki, Minoru Asada, Hiroki Mori

**Affiliations:** 1 Institute for Open and Transdisciplinary Research Initiatives, Osaka University, Suita, Osaka 565-0871, Japan; 2 Graduate School of Engineering, Osaka University, Suita, Osaka 565-0871, Japan; 3 Future Robotics Organization, Waseda University, Shinjuku, Tokyo 169-8555, Japan

**Keywords:** computational model, complexity, network structure, complex network theory, spiking neuron, self-organization

## Abstract

In this study, simulations are conducted using a network model to examine how the macroscopic network in the brain is related to the complexity of activity for each region. The network model is composed of multiple neuron groups, each of which consists of spiking neurons with different topological properties of a macroscopic network based on the Watts and Strogatz model. The complexity of spontaneous activity is analyzed using multiscale entropy, and the structural properties of the network are analyzed using complex network theory. Experimental results show that a macroscopic structure with high clustering and high degree centrality increases the firing rates of neurons in a neuron group and enhances intraconnections from the excitatory neurons to inhibitory neurons in a neuron group. As a result, the intensity of the specific frequency components of neural activity increases. This decreases the complexity of neural activity. Finally, we discuss the research relevance of the complexity of the brain activity.

## 1. Introduction

A macroscopic structure shapes its microscopic activity. A good building structure keeps intensity of reinforcing steels for a long time. Human society influences individual activities. What about a brain that has macroscopic anatomical structures and microscopic spiking activities?

The brain is a complex network composed of a large number of neurons and their connections, which are modified based on the activation of neurons [1,2]. Complex network theory shows that a macroscopic anatomical brain network, which is constructed based on the anatomical connections between brain regions, has unique structural properties that are similar to a small-world network [3]. A small-world network is characterized by a high clustering coefficient and low shortest path length. The clustering coefficient indicates the density of the number of the connections between nodes that are closed triangles in the network, and the shortest path length indicates the averaged shortest distance between arbitrary nodes (detailed in Section 2.3.3). The high clustering coefficient and low shortest path length in the small-world network enhance the local and global information transmission from one node to other nodes [4,5,6]. Therefore, the smallworldness of the brain network may allow for efficient information transmission and processing in local and global brain regions [7]. However, it is unclear how the structural properties of the small-world network affect the macroscopic activity of a brain region, the microscopic activities of individual neurons, and the functional network.

Complexity has been used to characterize the dynamics of biological signals, which have different dynamical properties at each time scale. In numerous cases, complexity refers to the unpredictability of time-series signals on multiple time scales based on sample entropy [8]; this is referred to as multiscale entropy (MSE) [9,10]. Studies on the autism spectrum disorder (ASD) suggested that the complexity of the brain activity is closely related to certain structural properties of the anatomical network. Bosl et al. [11] showed that the complexity of electroencephalogram (EEG) signals during the resting state of ASD children is lower than that of typically developing (TD) children in certain brain regions. Moreover, several studies suggested that the anatomical network in the brain of ASD has excessive connections in certain local regions [12] and the disconnection of the long-ranged shortcut path between regions [13] (i.e., high clustering coefficient and high shortest path length). In addition, these studies discussed that these kinds of atypical structures may cause different brain dynamics and cognitive functions of ASD [13,14].

Computational studies have shown the relationship between the complexity of a brain activity and an anatomical network. Friston [15] showed that the dimensional complexity of network activity decreases with an increase in the strength of the connections between neuron groups in a network model. Nakagawa et al. [16] showed that reduction in the strength of the connections between the groups of spiking neuron models reduces MSE at slow time scales. However, these studies did not consider any macroscopic network structures. Sporns et al. [17] optimized the connections between neuron models to maximize various information-theoretical measures (entropy, functional segregation, which is an indicator of the complexity of a systems, increases when a subnetwork within a network has independent activity from other subnetworks. This concept is different from MSE, which measures the unpredictability of a one-dimensional time-series signal. In this study, we employ MSE as a measure of complexity to consider the correspondence with existing EEG and magnetoencephalography (MEG) studies, and integration) of network activity and analyzed the relationship between a network structure and activity. In particular, they found that a small-world structure emerges when the functional segregation of network activity is maximized. However, the neural network models in the existing studies do not have a mechanism of neural plasticity. Synaptic connections can be modified by the activities of neurons, e.g., spike-timing-dependent plasticity (STDP) [2], in biological neural networks. Therefore, the neural activities may affect changes of macroscopic structural properties. Moreover, microscopic connectivity is modified by the synaptic plasticity. The complexity of neural activity may change owing to self-organization of macro and microscopic connectivities.

**Figure 1:**
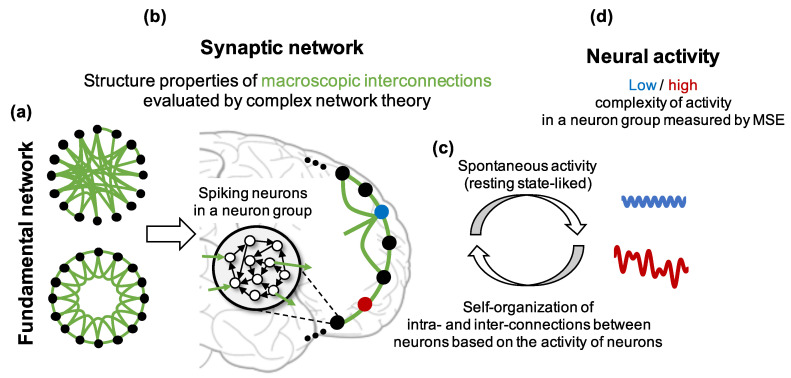
Hypothesis and assumptions about relationships among the fundamental network, synaptic network and the complexity of neural activity in this study. The black node and empty black circle represent a neuron group and a neuron in the group, respectively. The black and green lines indicate an intraconnection between neurons in a neuron group and an interconnection between neurons in different neuron groups, respectively. The red and blue dots show neuron groups with and without high clustering coefficient and high shortest path length, respectively.

In this study, we aim to comprehend how the structural properties of the macroscopic brain network influence the complexity of brain activity in each region using a spiking neural network model. Here, we control the clustering coefficient and path length of networks based on the Watts and Strogatz model (hereafter, WS model). The WS model can adjust them without changing the number of connections. This approach would enable us to identify dominant structural properties that may affect the complexity of brain activity. We hypothesize that a macroscopic network with high clustering coefficient and high shortest path length presents low complexity of brain activity in each region. Finally, we discuss the correspondence between our simulation results and existing ASD studies. The main procedures and analyses for verifying our hypothesis are as follows (see Figure 1):

1. Construct neuron groups consisting of spiking neurons that have weighted connections to randomly selected neurons in the same neuron group (intraconnections). We assumed that a neuron group and the intraconnections inside it correspond to a brain region and the intraconnections in the regions, respectively.
2. Determine the initial macroscopic network structure of neuron groups (fundamental network) based on the WS model. Then, if an edge exists between two neuron groups in the fundamental network, construct synaptic connections from the neurons in the group to the neurons in the other group (interconnections). We assumed that the average of interconnections between neuron groups correspond to the long-ranged interconnectivity between brain regions (synaptic network, Figure 1b).
3. Apply a plasticity rule to synaptic weights and self-organize the network (Figure 1c). If an edge does not exist between two neuron groups in the fundamental network, the weights between them remain zero to keep the given small-world structure.
4. Analyze the complexity of the activity and structural properties of the self-organized synaptic network using MSE and complex network theory to show their relationship (Figure 1d).
5. Investigate the frequency characteristics, firing rate, and intraconnections in each neuron group to explore the possible mechanisms of decrease in the complexity of neural activity.

## 2. Materials and Methods

### 2.1. Spiking Neural Network Model

**Figure 2:**
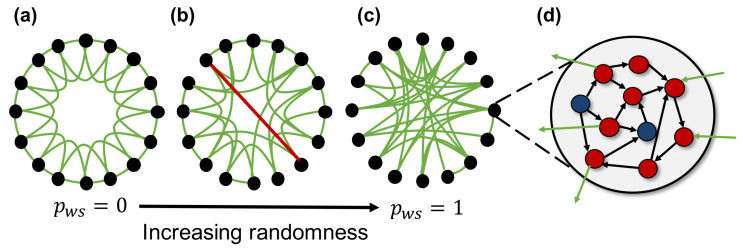
Overview of the spiking neural network model. A network is created using 100 neuron groups with macroscopic connections between neuron groups based on the Watts and Strogatz model [21]. The black nodes and green edge represent the neuron groups and macroscopic connections, respectively. (**a**) a lattice network where each node connected with neighboring nodes has local over-connectivity. All connections are rewired with rewiring probability $p_{WS}$, and larger $p_{WS}$ yields more random network; (**b**) a small-world network with a large number of clusters and shorter path length compared with other networks; (**c**) a random network where nodes are completely randomly connected to each other; (**d**) each neuron group contains 800 excitatory (red circles) and 200 inhibitory (blue circles) spiking neurons, and each neuron has intra- (black arrow) and inter-connections (green arrow).

Figure 2 shows an overview of the spiking neural network model used in this study. The network model consists of 100 neuron groups, and each neuron group contains 1000 spiking neurons. These neuron groups are macroscopically interconnected based on the macroscopic network constructed according to the WS model (fundamental network). A neuron in a neuron group is randomly intraconnected with the neurons in the same group (intraconnections) and interconnected with neurons of the neuron groups (interconnections) connected as the edges of the fundamental network. Each connection between neurons has a weight, which is modulated by activity-dependent plasticity. Therefore, the network self-organizes based on the neural activities. Here, we use an STDP [18] rule, which has been known as a biologically plausible rule [19], in order to update the weights of connections. Parameters of STDP are the same as those used in the previous study [20]. Hereafter, we assume that the average of the weighted interconnections between neurons corresponds to macroscopic connections between neuron groups (synaptic network).

#### 2.1.1. Neuron Model

In this study, we employed the Izhikevich spiking neuron model [22]. This model can represent various firing patterns (e.g., regular, bursting, and chattering), and their synchronization at various frequencies represents activity patterns. Furthermore, this model provides computational efficiency; therefore, a large network can be efficiently constructed. In this study, excitatory and inhibitory neurons are used to increase and suppress the firing of postsynaptic neurons, respectively. These parameters are the same as those used in the Izhikevich model [22].

#### 2.1.2. Construction of Fundamental Network Using the Watts and Strogatz Model

The initial interconnections in the synaptic network are determined by the edges of the fundamental network. The fundamental network is constructed by the WS model to change the structural properties of interconnections without changing the number of connections. The WS model is one method of constructing a small-world network, and it can adjust the clustering coefficient and average of shortest path length. The construction procedure of the fundamental network is as follows:

1. Construct a lattice (regular) network, where each neuron group is connected to *k* neighboring neuron groups (Figure 2a). the lattice network has numerous clusters and a long path length.
2. Randomly rewire each edge according to a rewiring probability, $p_{WS}$. This creates a shortcut between neuron groups, as shown by the red line in Figure 2b. The network structure becomes random, and the number of clusters and path length decrease as $p_{WS}$ increases (Figure 2c). The typical value of $p_{WS}$ used to construct the small-world network is between 0.01 and 0.1.

### 2.2. Parameters and Simulation Setting

Table 1 summarizes the model parameters used in this study. These parameters are determined based on previous studies [20,22,23]. The model consists of $N_{\mathrm{group}}$ (=100) neuron groups, and each neuron group contains $N_{E}$ (=800) excitatory neurons and $N_{I}$ (=200) inhibitory neurons. Here, an excitatory neuron is intraconnected with $C_{\mathrm{intra}}$ (=100) randomly selected neurons in the same group and interconnected with $C_{\mathrm{inter}}$ (=3) randomly selected neurons of the connected neuron group. An inhibitory neuron is intraconnected with $C_{\mathrm{intra}}$ (=100) randomly selected excitatory neurons in the same group; however, there are no interconnections for inhibitory neurons. $C_{\mathrm{intra}}$ and $C_{\mathrm{inter}}$ are fixed through simulation. $N_{group}$ and *k* (=6) are experimentally determined, so that a fundamental network shows difference in its structural properties according to rewiring probability $p_{WS}$. In this study, we used {0.0, 0.002, 0.005, 0.01, 0.02, 0.05, 0.1, 0.2, 0.3, 0.4, 0.5, 0.6, 0.7, 0.8, 0.9, 1.0} as the values of $p_{WS}$.

**Figure 3:**
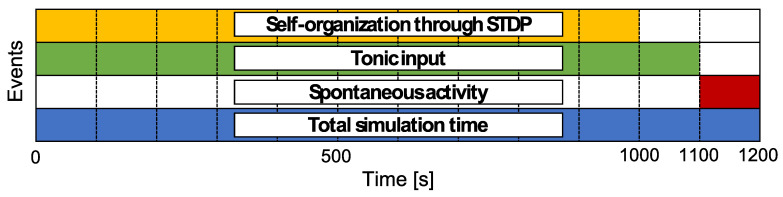
Time schedule for simulation. Each colored area indicates the period for which the event occurred. Neural activities during 1110 s to 1200 s were analyzed to determine the relationship among the structural properties of the synaptic network and neural activity.

The simulation schedule is shown in Figure 3. The total simulation time is 1200 s, and one time step is 1 ms. The duration for self-organization through STDP is set as 1000 s. Furthermore, the tonic input duration, $T_{tonic}$, is set as 1100 s to drive neural activity after self-organization. Then, the activity without any external inputs, like a resting state, during 1100 s to 1200 s is analyzed as the neural activity of the network model. The simulation is independently conducted ten times for each $p_{WS}$.

### 2.3. Analysis Method for Neural Activity and Network Structure

#### 2.3.1. Analysis Method for Complexity of Neural Activity

In this study, we utilize local averaged potential (LAP) [24] as the representative activity of a neuron group. LAP is the average of the membrane potentials of the excitatory neurons within a neuron group. The LAP is not directly equivalent to local field potential, which is recorded in the extracellular space around neurons, or to the EEG signals in the brain, which are typically used as an index for electric potentials. Nevertheless, LAP can directly reflect the collective activity and can be considered as an indicator of the synchronous activity of a neuron group.

MSE analysis was proposed by Costa et al. [9,10] to calculate the complexity (degree of irregularity) of biological series signals over multiple time scales. MSE can be obtained by calculating sample entropy, which shows the unpredictability of time-series signals, for each coarse-grained signal at multiple time scales. First, an original signal, x(t), is down sampled by multiple time scales to obtain coarse-grained signals, y(t). In this study, an LAP signal is used for x(t), and MSE is calculated for each neuron group:

(1) $$y\left(t\right)=\frac{1}{\epsilon }\sum_{i=(t-1)\epsilon +1}^{i=t\epsilon }x\left(i\right)\;\;\;\;\left(1\leq t\leq N/\epsilon \right),$$

where ϵ indicates the scale factor. Sample entropy is calculated using the following equation for each coarse-grained signal:

(2) $$SampEn\left(r,m,N\right)=-\ln[C_{m+1}\left(r\right)/C_{m}\left(r\right)],$$

(3) $$C_{m}\left(r\right)=\frac{\mathrm{number}\;\mathrm{of}\;\mathrm{pairs}\left(i,j\right)\;(|z_{i}^{m}-z_{j}^{m}|<r,\;i\neq j)}{\left(N-m+1)(N-m\right)},$$

where $z_{i}^{m}=\left\{y_{i},y_{i+1},\cdots ,y_{i+m-1}\right\}$ denotes a subsequence of the coarse-grained signals from the *i*th to the (i+m−1)th data point of y(t), *m* denotes the length of the subsequence, $Y=\{y_{1},\cdots ,y_{i},\cdots ,y_{N}\}$ denotes the coarse-grained signals, and *N* denotes the length of *Y*. Sample entropy evaluates the unpredictability of time-series signals as the logarithmic ratio of probabilities, $C_{m+1}\left(r\right)$ and $C_{m}\left(r\right)$. In this study, we use m=2 and r=0.15, which are commonly used for MSE analysis.

#### 2.3.2. Analysis Method for Neural Activation in Neuron Groups

We analyze the frequency characteristics of LAP signals to clarify the effect of the frequency components of neural activity on MSE values. LAP signals are decomposed into frequency components using the fast Fourier transform (FFT), and the peak frequency with the highest intensity is obtained as the robust frequency components in neuronal activity. In addition, we investigate the firing rates to elucidate how the microscopic activation of neurons affects the complexity of the activity in a neuron group (see Supplementary Materials).

#### 2.3.3. Complex Network Analyses of Structural Properties

To investigate how the structural properties of the fundamental network affect the self-organization of the network and the complexity of neural activity, we considered following features of the network using complex network theory: the graph to be analyzed is composed of 100 nodes, and each node corresponds to a neuron group. The average of weights of the interconnections of neurons between neuron groups is used as the weight of the edge between nodes. We evaluate the clustering coefficient, shortest path length, and degree centrality of the weighted directed networks.

- Clustering coefficient: The proportion of connections with the shape of a closed triplet over all possible combinations of triplets formed by three nodes in a network. This is defined as follows:
  (4) $$C_{i}=\frac{\mathrm{number}\;\mathrm{of}\;\mathrm{closed}\;\mathrm{triangles}}{\mathrm{number}\;\mathrm{of}\;\mathrm{possible}\;\mathrm{triangles}}.$$
  As we consider a directed weighted network, we use the algorithm of [25]. A node with a high clustering coefficient indicates that the node interacts with neighboring nodes more locally, and this may induce a synchronized behavior between nodes [5,6].
- Average shortest path length: the shortest path length is defined as the minimum number of steps required to pass from one node to another node in a network [26].
- Degree centrality: it refers to the number of connections of a node [27].

In the WS model, the average clustering coefficient and the average shortest path length in the network are decreased if $p_{WS}$ is increased. However, in the case of degree centrality, the average value is not changed but the variance is increased with $p_{WS}$. We show the relationship between these complex network measures and MSE. In addition, intraconnections are organized under inputs through interconnections, which are specified in accordance with the fundamental network. Hence, this self-organization of interconnections and intraconnections may affect the complexity of the activity of a neuron group. Therefore, we investigate the average intraconnection in a neuron group and examine its relationship with the structural properties and MSE of activity for each neuron group after self-organization.

## 3. Results

In our model, the self-organized synaptic network showed spontaneous activity even after stopping the tonic input. However, the network did not show spontaneous activity if we did not use STDP for self-organization; these were also reported in previous studies [20,28]. We assumed that the spontaneous activity corresponds to the brain activity in the resting state. Hereafter, we mainly analyze this spontaneous activity.

We present the result of the analyses in this section. The main results are as follows:

1. The analysis of MSE showed that each neuron group had different levels of the complexity of neural activity and the average complexity of all neuron groups decreased if the fundamental network had small $p_{WS}$ (Section 3.1).
2. The analysis of neural activity in neuron groups with different values of MSE showed that a neuron group with low complexity exhibited increased signal amplitude in two frequency bands (20–40 and 40–60 Hz) of neural activity (Section 3.2).
3. The complex network analyses for each neuron group showed that the local over-connectivity (the clustering coefficient and degree centrality were high) and complexity of a neuron group had a negative relationship (Section 3.3).

### 3.1. Relationship between MSE and WS Model

**Figure 4:**
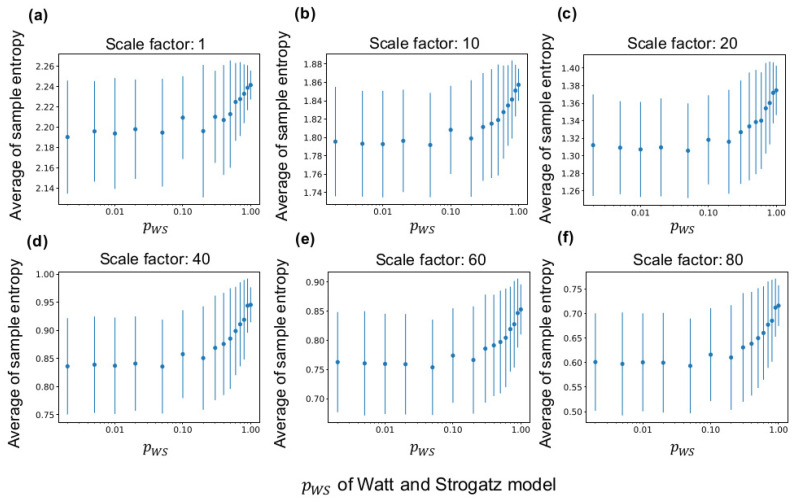
Relationship between sample entropy and $p_{\mathrm{WS}}$ of the WS model. The *x*-axis in all graphs represents $p_{\mathrm{WS}}$. (**a**–**f**) average sample entropy of all neuron groups with ten independent simulations at scale factors (ϵ in Equation (1)) of multiscale entropy (MSE) at 1, 10, 20, 40, 60, and 80. The error bars indicate the standard deviation.

**Figure A1:**
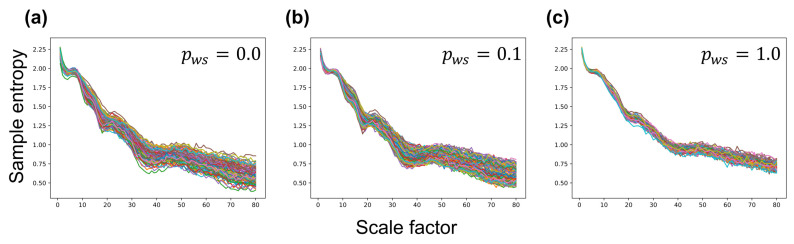
Multiscale entropy (MSE)-based complexity curves of each neuron group in a synaptic network. (**a**) lattice network ($p_{\mathrm{WS}}=0.0$); (**b**) a small-world network ($p_{\mathrm{WS}}=0.1$); (**c**) a random network ($p_{\mathrm{WS}}=1.0$). The *y*-axis indicates sample entropy, and the *x*-axis indicates scale factor ϵ.

Figure 4 shows the sample entropy of a LAP signal according to $p_{\mathrm{WS}}$ of the fundamental network (see Section 2.3.1 for the method). According to the figure, the average of sample entropy of all neuron groups increases with $p_{\mathrm{WS}}$ at any time scales and decreases at large scale factors of MSE (see Figure A1 for curves of sample entropy on all scale factors). Therefore, the average of sample entropy decreases if the clustering coefficient and shortest path length are high. Moreover, as the down sampling in Equation (1) acts like a low-pass filter, the complexity of the low frequency component appears to be smaller than that of the high frequency component. Furthermore, as shown in the figure, the average sample entropy of the synaptic network shows variance even when the same fundamental network is used for the lattice network. Therefore, the interconnections and neural activities in several neuron groups differ from those in the initial network because of self-organization.

### 3.2. Neural Activities in Neuron Groups with Different Levels of Complexity

**Figure 5:**
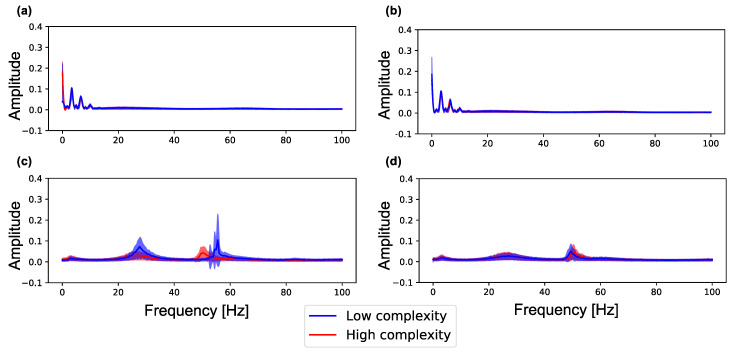
Amplitude of each frequency spectrum sampled from the local averaged potentials of the 10 neuron groups with low complexity (blue) and high complexity (red) in a network. The peak envelopes is used to plot the curve in the figure. Color curves and color-shaded areas represent average and standard deviation values for ten simulations, respectively. (**a**) the lattice network ($p_{\mathrm{WS}}=0.0$) during self-organization by spike-timing-dependent plasticity (STDP) (0–100 s); (**b**) the random network ($p_{\mathrm{WS}}=1.0$) during self-organization by STDP (0–100 s); (**c**) the lattice network ($p_{\mathrm{WS}}=0.0$) after self-organization by STDP (1100–1200 s); (**d**) the random network ($p_{\mathrm{WS}}=1.0$) after self-organization by STDP (1100–1200 s).

**Figure A2:**
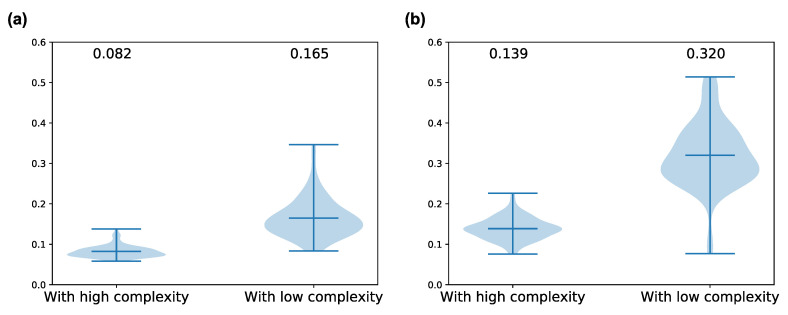
The differences of peak amplitude of spontaneous neural activity in some frequency bands between neuron groups with low and high complexity when $p_{WS}=0.0$. We used 10 neuron groups with high and low complexity in each simulation as comparison data. The number on above each violin plot denotes the average value for ten simulations. Wilcoxon signed-rank test was used for statistical test. (**a**) amplitude in the 20–40 Hz band (Wilcoxon signed-rank test, statistic = 6.0, *p*-value = 4.6706 × $10^{-18}$); (**b**) amplitude in the 40–60 Hz band (Wilcoxon signed-rank test, statistic = 11.0, *p*-value = 5.4302 × $10^{-18}$).

Figure 5 shows the spectra in neuron groups with high and low complexity (summation of MSE for 80 scale factors) during (0–100 s) and after self-organization (1100–1200 s) in a lattice and random network. As shown in Figure 5a,b, neuron groups in the lattice and random networks show similar frequency distributions. However, as shown in Figure 5c, after self-organization, the neuron groups with low complexity in the lattice exhibit increased signal amplitude in the two frequency bands (20–40 and 40–60 Hz) as compared with the neuron groups with high complexity in the network (see Figure A2 for data distribution and statistical differences). On the other hand, as shown in Figure 5d, both neuron groups in the random network also show increased signal amplitude but show similar curves to the neuron groups with high complexity in the lattice network. Therefore, neuron groups in the random network, which has higher average and lower standard deviation of sample entropy than the lattice network (see Figure 4), have similar frequency properties to the neuron group with high complexity in the lattice network. This result indicates that the initial macroscopic network structure affects the self-organization of synaptic connections, resulting in neural activities in different frequency bands.

We show the relationship between the complexity and peak frequency of neural activity in all neuron groups in Figure A4. The figure shows the peak frequency and their amplitude in the three frequency bands (0–20, 20–40 and 40–60 Hz) in which many changes occurred in Figure 5. We observed the same tendency with Figure 5 that amplitude increases as the complexity of neural activity decreases in 20–40 Hz and 40–60 Hz bands.

### 3.3. Relationship between Neural Activity and Structural Properties

**Figure 6:**
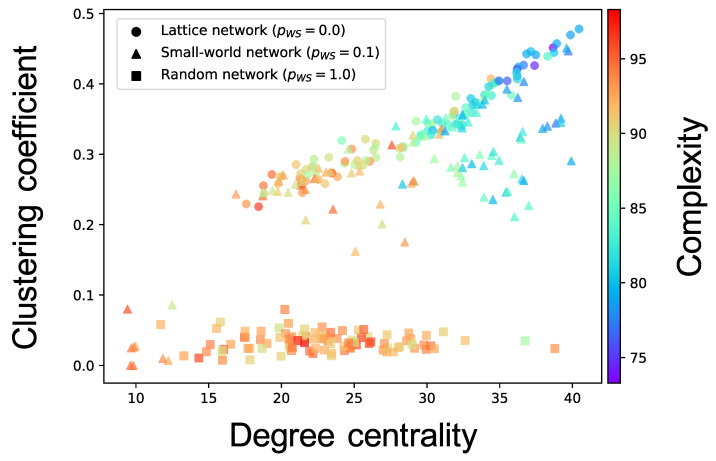
Relationship between the connectivity structure and complexity of neural activity. Each marker corresponds to a neuron group in the network, and its color indicates the summation of the sample entropy for all 80 scale factors. The *x*-axis indicates the degree centrality, and the *y*-axis indicates the clustering coefficient.

**Figure A3:**
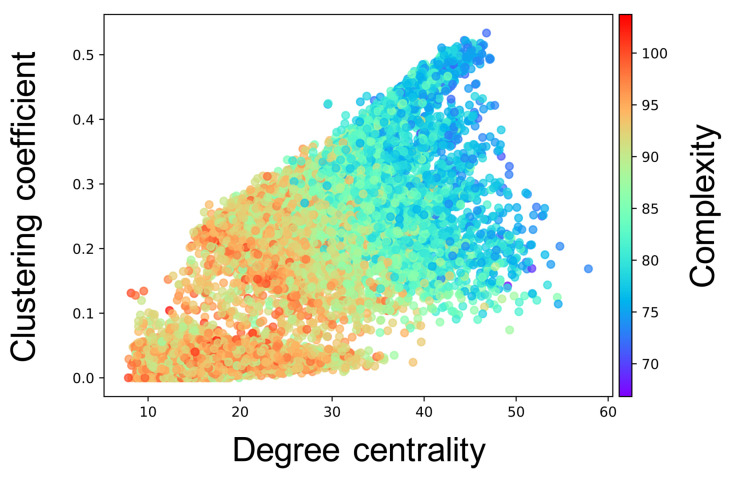
Relationship between the connectivity structure and the complexity of neural activity. Each marker corresponds to a neuron group in the network, and its color indicates the summation of the sample entropy for all 80 scale factors. The *x*-axis indicates the degree centrality, and the *y*-axis indicates the clustering coefficient.

**Figure A4:**
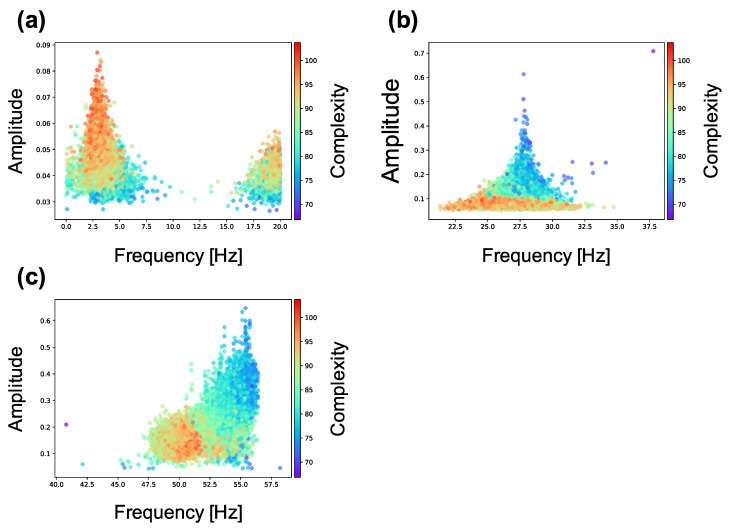
Relationship between the peak frequency and the complexity of neural activity. (**a**) relationship in the 0–20 Hz band; (**b**) relationship in the 20–40 Hz band; (**c**) relationship in the 40–60 Hz band. Each marker corresponds to a neuron group in the network, and its color indicates the summation of the sample entropy for all 80 scale factors. The *x*-axis indicates the peak frequency of the neural activity, and the *y*-axis indicates the amplitude. Figure shows that the amplitude in increases as the complexity of neural activity decreases in 20–40 Hz and 40–60 Hz bands. Furthermore, the peak frequency increases as the complexity of neural activity decreases in the 40–60 Hz band. However, amplitude decreases as the complexity of neural activity decreases in the 0–20 Hz band. This result indicates that the robust frequency components in neural activity of the neuron groups with low complexity shifted to the high frequency bands (20–40 Hz and 40–60Hz) from the low frequency bands (0–20 Hz).

**Figure A5:**
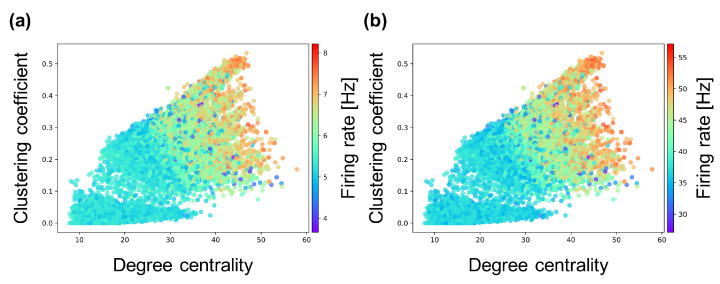
Relationship between the connectivity structure and the firing rate of excitatory and inhibitory neurons. Each marker corresponds to a neuron group in the network, and its color indicates the average firing rate of excitatory and inhibitory neurons. The *x*-axis indicates the degree centrality, and the *y*-axis indicates the clustering coefficient. (**a**) relationship between structural properties and firing rate of excitatory neurons; (**b**) relationship between structural properties and firing rate of inhibitory neurons.

**Figure A6:**
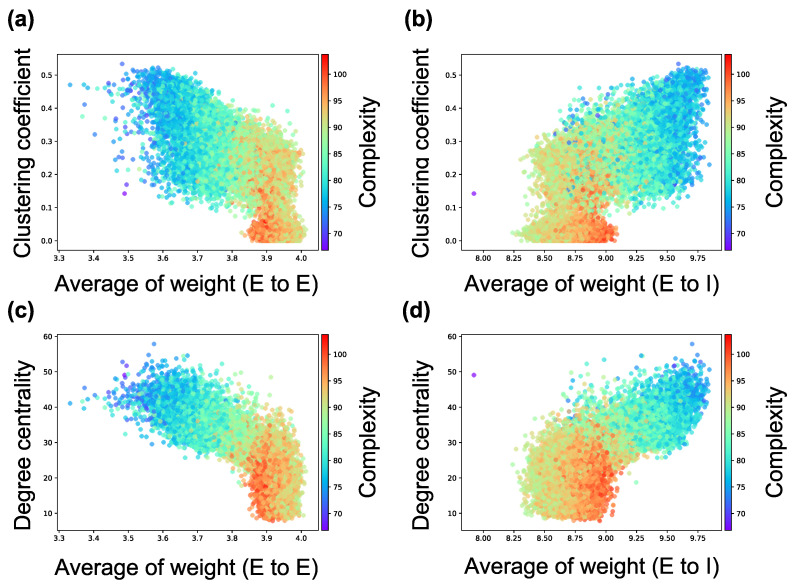
Relationship among the weight of intraconnection, structural properties, and complexity of neural activity. (**a**) relationship among the weight of intraconnection from excitatory to excitatory neuron, clustering coefficient based on the interconnection, and complexity; (**b**) relationship among the weight of intraconnection from excitatory to inhibitory neuron, clustering coefficient based on the interconnection, and complexity; (**c**) relationship among the weight of intraconnection from excitatory to excitatory neuron, degree centrality based on the interconnection, and complexity; (**d**) relationship among the weight of intraconnection from excitatory to inhibitory neuron, degree centrality based on the interconnection, and complexity. Each marker corresponds to a neuron group in the network, and its color indicates the summation of the sample entropy for all 80 scale factors. The *x*-axis indicates the average of weight of interconnection, and the *y*-axis the structural properties of interconnection.

**Figure A7:**
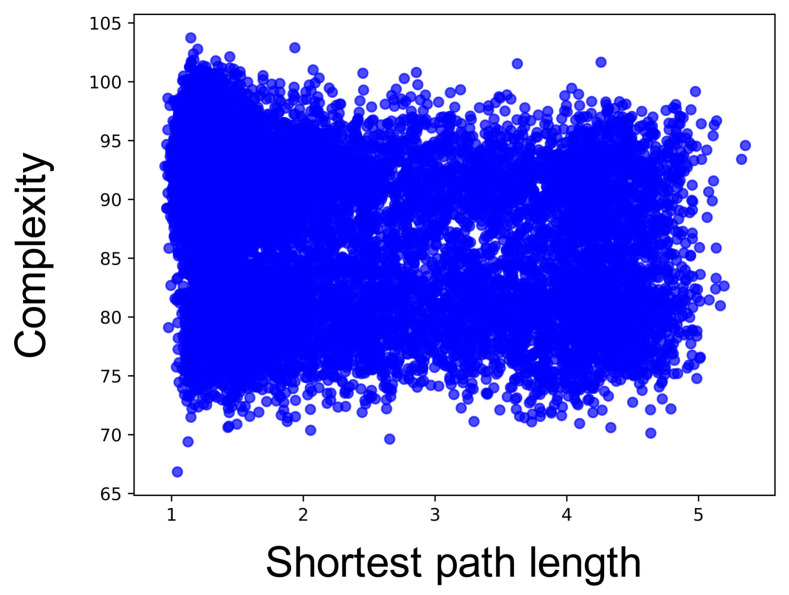
Relationship between shortest path length and complexity for each neuron group. The *x*-axis is the shortest path length, and the *y*-axis is the summation of the sample entropy for all 80 scale factors.

**Figure A8:**
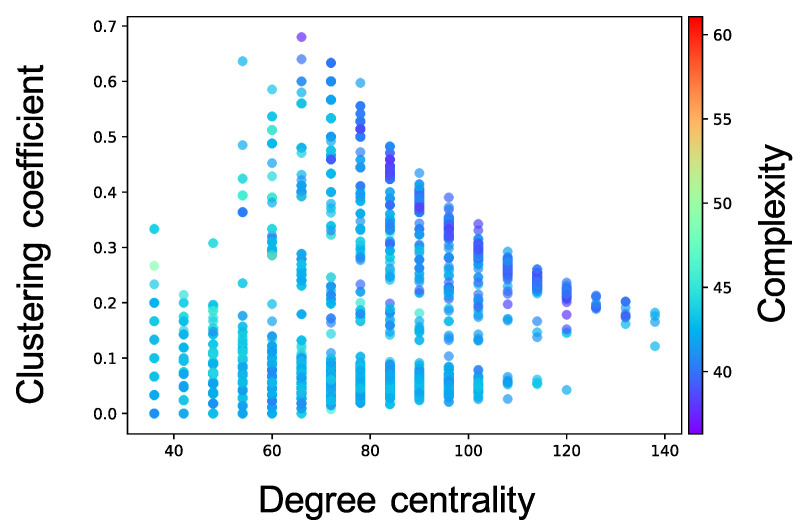
Relationship between the connectivity structure without STDP and the complexity of neural activity with the tonic input. Duration for tonic input was set as 100 s. Here, we used the same initial weights of the synaptic networks in Figure A3 and fixed the weights during the tonic input. Each marker corresponds to a neuron group in the network, and its color indicates the summation of the sample entropy for all 80 scale factors. The *x*-axis indicates the degree centrality, and the *y*-axis indicates the clustering coefficient.

Complex network analyses were performed for each neuron group after self-organization to clarify the factor that induced different values of the complexity of neural activity among neuron groups (see Section 2.3.3 for the method). Here, we show the results only for the cases where the $p_{\mathrm{WS}}$ of the WS model is 0.0, 0.1, and 1.0 of one simulation; the results for all values of $p_{\mathrm{WS}}$ of ten simulations are shown in Figure A3, Figure A4, Figure A5, Figure A6 and Figure A7. Figure 6 shows the relationship between the structural properties and complexity of neural activity for each neuron group in the synaptic network. As shown in this figure, the complexity of neural activity tends to decrease with increase in the clustering coefficient and degree centrality. As the cluster organizations of nodes originate from the lattice network in the WS model, they are constructed by neighboring (or local) neuron groups. Hereafter, we refer to the structural property in which the clustering coefficient and degree centrality are large as local over-connectivity (in the WS model, local-over connectivity is high when $p_{WS}$ is close to 0.0). In contrast, we could not find a relationship between the shortest path length and complexity (see Figure A7). We found no clear relationship between complexity of neural activity with tonic input (i.e., neural activities in the network are induced by external input instead of spontaneous activation) and structural properties of synaptic network without STDP compared to the activity after self-organization by STDP (see Appendix B and Figure A8).

**Figure 7:**
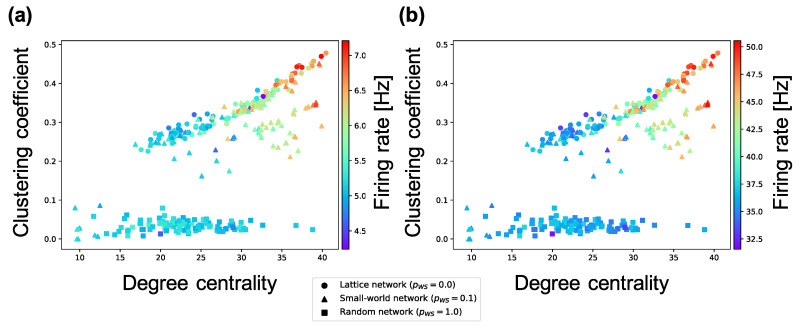
Relationship between the connectivity structure and firing rate of excitatory and inhibitory neurons. Each marker corresponds to a neuron group in the network, and its color indicates the average firing rate of excitatory and inhibitory neurons. The *x*-axis indicates the degree centrality, and the *y*-axis indicates the clustering coefficient. (**a**) relationship between structural properties and firing rate of excitatory neurons; (**b**) relationship between structural properties and firing rate of inhibitory neurons.

**Figure 8:**
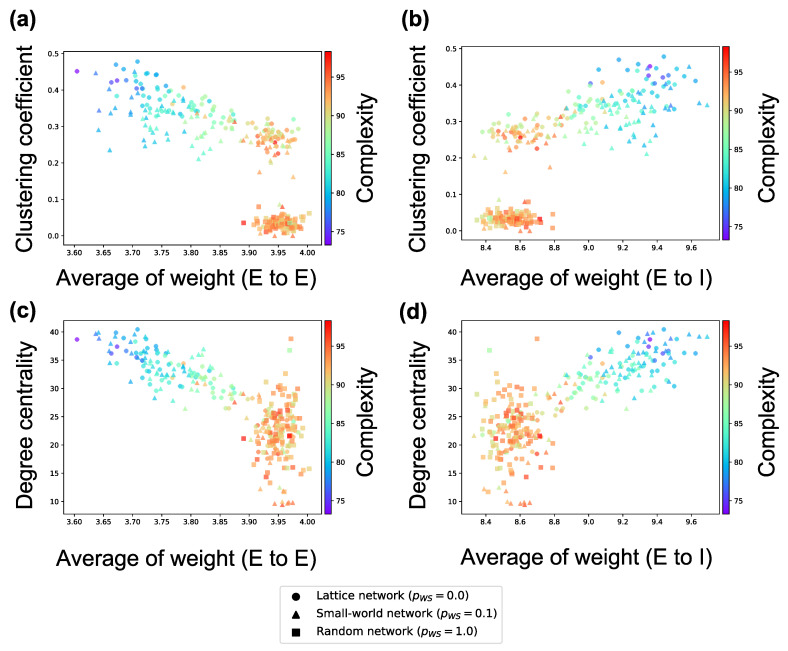
Relationship among the weight of intraconnection, structural properties of the synaptic network, and complexity of neural activity. (**a**) relationship among the average weight of intraconnections from excitatory neurons to excitatory neurons, clustering coefficient based on the interconnection, and complexity; (**b**) relationship among the average weight of intraconnections from excitatory neurons to inhibitory neurons, clustering coefficient based on the interconnection, and complexity; (**c**) relationship among the average weight of intraconnections from excitatory neurons to excitatory neurons, degree centrality based on the interconnection, and complexity; (**d**) relationship among the average weight of intraconnections from excitatory neurons to inhibitory neurons, degree centrality based on the interconnection, and complexity. Each marker corresponds to a neuron group in the network, and its color indicates the summation of the sample entropy for all 80 scale factors. The *x*-axis indicates the average weight of interconnection, and the *y*-axis the structural properties of interconnection.

The analyses performed until now targeted *inter*connections. Next, we investigate the average weight of *intra*connections in a neuron group and the average firing rates of neurons. According to Figure 7, the firing rates of excitatory neurons and inhibitory neurons in a neuron group increase in local over-connectivity. Furthermore, as shown in Figure 8, the average of the weights of the intraconnections from one excitatory neuron to another excitatory (inhibitory) neuron had a positive (negative) relationship with the average of the sample entropy and negative (positive) relationship with the local over-connectivity of the synaptic network. That is, the neuron groups with low complexity with local over-connectivity have small intraconnections from excitatory to excitatory neurons and large intraconnections from excitatory to inhibitory neurons, and they have increased firing rates of both types of neurons. Since the weights of connections from the inhibitory neurons to the excitatory ones had not changed by STDP, we omitted the results about the weights.

## 4. Discussion

We constructed a network model consisting of multiple spiking neuron groups to investigate how the macroscopic (inter-neuron groups) fundamental network structure influences its self-organization of the microscopic (synaptic) network and its activity from the perspective of the complexity of neural activity for each neuron group. We only controlled the structural properties of the macroscopic fundamental network using the rewiring probability of the WS model. Our simulation showed that self-organization under the macroscopic structure led to a change in the intraconnections in a neuron group, and, therefore, the complexity of neural activity decreased. Our complex network analyses implied that a higher clustering coefficient and degree centrality, which are indicators of the degree of local over-connectivity, might result in lower complexity.

### 4.1. Hypothetical Mechanism of Low Complexity because of Local Over-Connectivity

As shown in Figure 6, the complexity of neural activity in a neuron group decreases with the degree of local over-connectivity, i.e., the clustering coefficient and degree centrality. We suppose that this result is because the intraconnections change along with self-organization under the macrostructure. As shown in Figure 8, a neuron group with low complexity of neural activity shows increased average weights of intraconnections from excitatory to inhibitory neurons and decreased average weights of intraconnections between excitatory neurons. This indicates that a microstructure that suppresses the activity of excitatory neurons appear as a result of self-organization under local over-connectivity in the macroscopic network. However, as shown in Figure 7, the firing rates of excitatory and inhibitory neurons with the low complexity increase, i.e., a neuron group takes excessive input from other neuron groups. We suppose that the intraconnections might be self-organized through STDP to maintain a certain amount of neural activity (homeostasis) against excessive input from the other neuron groups [29]. As a result of self-organization, the strength of intraconnections from excitatory neurons to inhibitory neurons increases, which might cause oscillation of neural activities with two peaks within frequency bands (see Figure 5). Several studies have reported that inhibitory activation contributes to the induction of periodic patterns in brain activity [30,31].

Based on these results, we hypothesize a possible mechanism of the reduction in the complexity of neural activity in the synaptic network with local over-connectivity, as follows:

1. The firing rates of neurons in a neuron group with local over-connectivity increases because of excessive input from the connected neuron groups (see Figure 7). As a result, to maintain a certain amount of the activities of neurons, strengthened intraconnections from excitatory to inhibitory neurons and weakened intraconnections between excitatory neurons are self-organized by STDP (see Figure 8).
2. The increase in the strength of intraconnections to inhibitory neurons induces the oscillation of excitatory neurons, which increases the intensity of the several specific frequency components of neural activity (see Figure 5).
3. The signals of specific frequency components become robust. As a result, complexity decreases (Figure 6).

### 4.2. Relationship with Studies on ASD

In the current study, we found that the complexity of neural activity in a neuron group with local over-connectivity in the synaptic network decreases. This result aligns with the lower complexity of the EEG signals of ASD children (2 to 24 months) [11]. Therefore, in our model, the ASD-like low complexity of neural activity occurs when a neuron group in the synaptic network has local over-connectivity, as shown in Figure 6. Ghanbari et al. [32] showed that the complexity of MEG signals in several regions of the brain of ASD children (6–15 years) increased. We suppose that this discrepancy may be related to developmental changes in the anatomical network structure. Solso et al. [12] showed that over-connectivity was mainly found in the extremely early stages of development; this was not observed in ASD children aged 3–4 years. Therefore, our model shows the possibility that the local over-connectivity decreases the complexity of neural activity in ASD children aged 2–24 months. However, in the case of children aged 6–15 years, who were the research targets in the study of Ghabari et al. [32], we estimate that fluctuations in the complexity of brain activity in ASD may occur owing to factors other than local over-connectivity. Future studies may clarify the exact relationship between the structure of the anatomical network and the brain activity in ASD using a computational model that includes the developmental changes in the anatomical network structure with time.

### 4.3. Limitations and Future Work

Several limitations of this study should be acknowledged. A fundamental network was constructed based on the WS model to focus on the topological structure of a macroscopic network. However, it is well known that the anatomical network is a scale-free [33] network, which has a heavy-tailed distribution of the degree of nodes, or rich-club networks [34], which have connections between the nodes that have high degree centrality. As shown in Figure 6, our results show that the degree centrality in a synaptic network is an important factor for inducing different complexities of neural activity. Investigating neural activities when such network structures are used as a fundamental network is an interesting point. In addition, we did not consider distance between neuron groups in the macroscopic network. Considering the relative distance between neuron groups which may relate to information transmission delay is also an interesting topic [35]. Moreover, in our study, a randomly connected structure was used for the neuron groups in the network model; however, it is well known that the cortex has various laminar structures. Furthermore, numerous studies on ASD have suggested that the atypical balance of excitatory to inhibitory neurons in the cortex induces altered neural connectivity and activity in brains with ASD [36,37,38]. In the future, we should consider changing these kinds of parameters or structures. This approach would provide a deep understanding about the relationship between the structure and activity of the brain, at least for a few areas of the brain.

In the current model, we have not yet introduced external input or attached a body because the purpose of our research was to understand the relationship between the brain structures related to the complexity of spontaneous activity in the brain. However, the brains of humans and animals perceive sensory signals and exhibit behaviors throughout the body. How the brain network is self-organized through interactions with the environment, and how it influences behaviors are cutting-edge topics of research in developmental science. Several studies have used computational modelling and shown the importance of the interaction between the body and brain to exhibit diverse behavior [39,40], to self-organize the brain [41], or to learn a task [42,43]. Furthermore, several studies have shown the low complexity of brain activity or the abnormal structure of a functional network during a task [44] while watching a video [45] or based on the video-EEG data [46]. It would be interesting to understand how different anatomical network structures influence the flow of external information from between different regions of a network. This would highly enrich this approach from the view of informational theory. In any case, we believe that this study could help in achieving deeper understanding of the mechanisms underlying the atypical behavior or brain activity of ASD by examining how neural activity or behavior may change by connecting a body or external input to a physical network.

## 5. Conclusions

The purpose of this study was to understand how the structure of a macroscopic network structure relates to the microscopic activity of a spiking neural network. In particular, we hypothesized that the structural properties of a macroscopic anatomical brain network would induce lower complexity of brain activity. To address this issue, we constructed a neural network using multiple neuron groups that consisted of spiking neurons, and changed the clustering coefficient and shortest path length in the network using the WS model. Then, we analyzed their spontaneous activity based on MSE. The results of complex network theory analysis and neural activity for each neuron group in a synaptic network showed that the local over-connectivity in the synaptic network decreased complexity and enhanced the intensity of specific frequency components of brain activity.

Based on the experimental results, we proposed the hypothetical mechanism that a neuron group with local over-connectivity self-organizes the intraconnections to maintain a certain amount of neural activity against excessive external input from the connected neuron groups. As a result of the hypothesis, the intensity of the specific frequency components of neural activity increases and, therefore, the complexity of neural activity decreases.

## Figures and Tables

**Table 1:** Parameters of the simulation model used in this study.

Parameters	Values	Descriptions	Notes
$D_{\mathrm{intra},\mathrm{exc}}$	[0, 20]	Transfer delay of excitatory synapse in neuron group	(uniform dist., ms)
$D_{\mathrm{intra},\mathrm{inh}}$	1	Transfer delay of inhibitory synapse in neuron group	(ms)
$w_{\mathrm{init},\mathrm{exc}}$	6.0	Initial weight of excitatory synapse	-
$w_{\mathrm{init},\mathrm{inh}}$	−5.0	Initial weight of inhibitory synapse	-
$w_{\mathrm{upper}}$	10.0	Maximum value of weight	-
$N_{E}$	800	Number of excitatory neurons in a neuron group	-
$N_{I}$	200	Number of inhibitory neurons in a neuron group	-
*N*	1000	Number of neurons in a neuron group	$=N_{E}+N_{I}$
$C_{\mathrm{intra}}$	100	Number of intraconnections of a neuron	-
$D_{\mathrm{inter},\mathrm{exc}}$	[10, 30]	Transfer delay of excitatory synapse between neuron groups	(uniform dist., ms)
$N_{\mathrm{group}}$	100	Number of neuron groups	-
*k*	6	Number of edges for each neuron group	-
$C_{\mathrm{inter}}$	3	Number of interconnections of a excitatory neuron	-
$p_{\mathrm{WS}}$	[0.0, 1.0]	Rewiring probability	-
$t_{\mathrm{step}}$	1	Time step	(ms)
$T_{\mathrm{total}}$	1200	Total simulation time	(s)
$T_{\mathrm{tonic}}$	1100	Time length of tonic input	(s)
$T_{\mathrm{STDP}}$	1000	Time length of self-organization through STDP	(s)
$N_{\mathrm{sim}}$	10	Number of independent simulations	-

## Supplementary Materials

The following are available online at https://www.mdpi.com/1099-4300/21/2/214/s1: values of complexity, structural properties of synaptic network, peak frequency and peak amplitude of LAP; Values of MSE for 80 scales.

## Appendix B. Complexity of Neural Activity without STDP

We conduct experiments of the complexity index for the network before the self-organization by STDP to clarify the role of the self-organization while no spontaneous activity without tonic input occurs before the self-organization. We analyze the activity with all network structures that we adopted above with the random tonic inputs and the fixed initial weights. Figure A8 shows the result of complexity index with clustering coefficient and degree centrality. There is no clear relationship between the complexity and structural properties of synaptic network compared to Figure A3. Therefore, the relationship between structural properties and complexity must be induced by the self-organization under the macroscopic structure through STDP.
